# Ex-vivo mechanical sealing properties and toxicity of a bioadhesive patch as sealing system for fetal membrane iatrogenic defects

**DOI:** 10.1038/s41598-020-75242-y

**Published:** 2020-10-29

**Authors:** Talita Micheletti, Elisenda Eixarch, Sergio Berdun, Germán Febas, Edoardo Mazza, Salvador Borrós, Eduard Gratacos

**Affiliations:** 1 BCNatal | Fetal Medicine Research Center (Hospital Clínic and Hospital Sant Joan de Déu), University of Barcelona, Building Helios 2, Sabino Arana Street, 1, 08028 Barcelona, Spain; 2 Institut d’Investigacions Biomèdiques August Pi i Sunyer (IDIBAPS), Barcelona, Spain; 3 Centre for Biomedical Research on Rare Diseases (CIBER-ER), Madrid, Spain; 4 Grup d’Enginyeria de Materials (GEMAT), Institut Químic de Sarrià, Universitat Ramon Llull, Barcelona, Spain; 5 Swiss Federal Institute of Technology, Zurich, Switzerland; 6 Empa, Materials Science and Technology, Dübendorf, Switzerland; 7 Institut de Recerca Sant Joan de Déu, Esplugues de Llobregat, Spain

**Keywords:** Biomedical materials, Bioinspired materials, Biomedical materials, Bioinspired materials, Surgery

## Abstract

Preterm prelabor rupture of membranes (PPROM) is the most frequent complication of fetal surgery. Strategies to seal the membrane defect created by fetoscopy aiming to reduce the occurrence of PPROM have been attempted with little success. The objective of this study was to evaluate the ex-vivo mechanical sealing properties and toxicity of four different bioadhesives integrated in semi-rigid patches for fetal membranes. We performed and ex-vivo study using term human fetal membranes to compare the four integrated patches composed of silicone or silicone-polyurethane combined with dopaminated-hyaluronic acid or hydroxypropyl methylcellulose (HPMC). For mechanical sealing properties, membranes were mounted in a multiaxial inflation device with saline, perforated and sealed with the 4 combinations. We measured bursting pressure and maximum pressure free of leakage (n = 8). For toxicity, an organ culture of membranes sealed with the patches was used to measure pyknotic index (PI) and lactate dehydrogenase (LDH) concentration (n = 5). All bioadhesives achieved appropriate bursting pressures, but only HPMC forms achieved high maximum pressures free of leakage. Concerning toxicity, bioadhesives showed low PI and LDH levels, suggesting no cell toxicity. We conclude that a semi-rigid patch coated with HPMC achieved ex-vivo sealing of iatrogenic defects in fetal membranes with no signs of cell toxicity. These results warrant further research addressing long-term adhesiveness and feasibility as a sealing system for fetoscopy.

## Introduction

Fetoscopic surgery is used in thousands of pregnancies worldwide yearly for a variety of fetal indications^1^. One of the main unsolved drawbacks of fetoscopy is the damage created to fetal membranes, which is thought to explain that iatrogenic preterm prelabor rupture of membranes (PPROM) occurs in up to 30% of cases^1,1^. PPROM is the main complication after fetoscopy and its occurrence substantially increases the risk of preterm birth and perinatal morbidity^1,1^.

In most instances, PPROM occurs weeks after fetoscopy^4^. Previous studies have shown that human fetal membranes do not heal after the creation of an iatrogenic defect^5–9^. However, it has also been shown how the natural sliding of the amnion over the chorion might provide a natural protective mechanism against PPROM^6^, which may explain why only a very small fraction of patients have PPROM within days of the procedure. On the other hand, mechanical factors such as chorioamniotic membrane separation after fetoscopy^10,10^ or septostomy^12^ increase the risk of PPROM weeks later. This suggests that mechanical factors interfering with the adhesion between chorion and decidua may be an important trigger eventually leading to PPROM.

Several systems have been attempted to seal fetal membranes, mainly in ex-vivo studies, so far without success. The injection of fluid adhesives, such as mussel-glues^13,13^ and medical sealants^15–17^ needs to overcome the problem of applying a fluid adhesive in the amniotic fluid wet environment. The use of collagen plugs across the membrane defect has shown poor results when tested in clinical conditions^18,18^. We hypothesized that a semi-rigid disc patch coated with bioadhesives could seal the amniotic defect, preventing exposure of the chorion to amniotic fluid and reducing the risks of chorion-decidua separation. To this end, we designed a semi-rigid patch made up of silicone, which can be coated with different combinations of bioadhesives.

In this study we tested the proof of concept that this integrated semi-rigid disc bioadhesive patch could achieve effective adhesion properties in human fetal membranes similar to well known adhesives, such as cyanoacrylate glue, but without their limitations to intrauterine use.

## Results

### Patients

Fetal membranes from a total of 27 donors were collected, 22 for mechanical tests and 5 for toxicity tests. Gestational age at delivery was 39.1 weeks (range 39–39.4) and mean birth weight was 3213.6 g ± 457.9. Indications for cesarean section were previous C-section (59.3%), breech presentation (29.6%) and more uncommonly placenta previa, pelvic tumors and previous pelvic floor surgery.

### Bioadhesives

**Figure 1:**
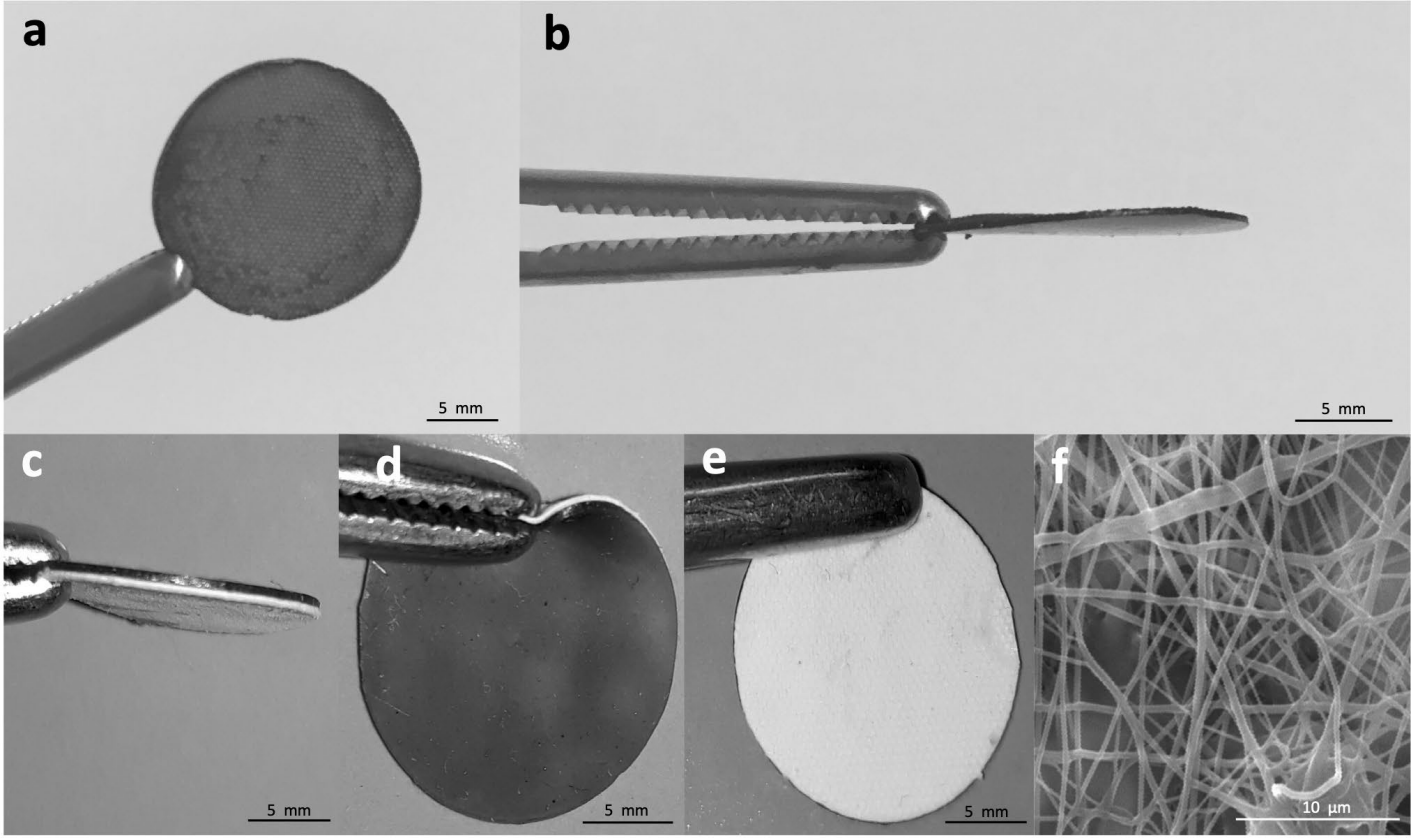
Semi-rigid patches composed of silicone only or silicone combined with electrospun polyurethane. (**a**,**b**) Silicone patch coated with dopaminated hyaluronic acid (DHA) in anterior and lateral views. (**c**–**e**) Silicone with electrospun polyurethane patch in lateral, posterior and anterior views. (**f**) Detail: electrospun polyurethane.

Four different bioadhesive-coated patches were developed, by combining a semi-rigid patch and a bioadhesive component. The candidates were composed of silicone-dopaminated hyaluronic acid (S-DHA), silicone with polyurethane-dopaminated hyaluronic acid (SPU-DHA), silicone-hydroxypropyl methylcellulose (S-HPMC) or silicone with polyurethane-hydroxypropyl methylcellulose (SPU-HPMC), as indicated in Table 1 and shown in Fig. 1.

**Table 1:** Types of sealing systems and composition.

Type of patch	Abbreviation	Semi-rigid patch	Bioadhesive component
Silicone-DHA	S-DHA	Medical silicone disc (450 μm-thick and 17 mm-diameter)	DHA 70–80% of dopamination, 10 mg/mL
Silicone–polyurethane-DHA	SPU-DHA	Medical silicone with electrospun polyurethane disc (480 μm-thick and 17 mm-diameter)	
Silicone-HPMC	S-HPMC	Medical silicone disc (450 μm-thick and 17 mm-diameter)	HPMC 300 μL, 10 mg/ml
Silicone–polyurethane-HPMC	SPU-HPMC	Medical silicone with electrospun polyurethane disc (480 μm-thick and 17 mm-diameter)	

DHA, dopaminated hyaluronic acid; HPMC, hydroxypropyl methylcellulose; S, silicone; SPU, silicone-polyurethane.

### Mechanical properties

As expected, bursting pressures after perforation and sealing were much lower than those observed in intact membranes (intact vs. any adhesive, *p* < 0.05).

**Figure 2:**
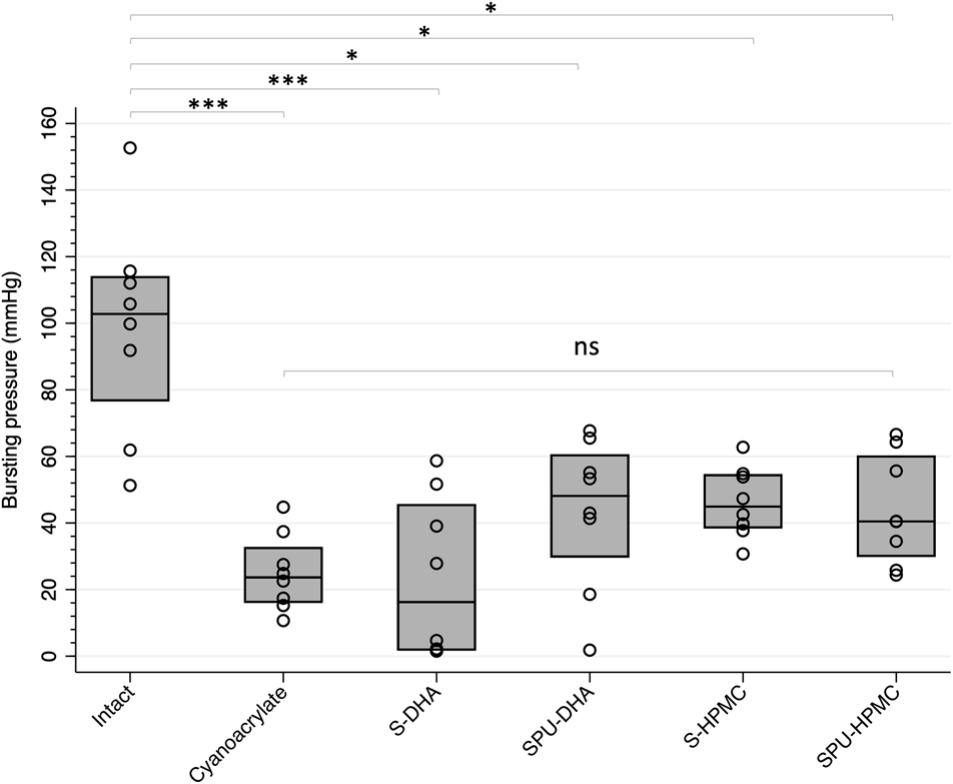
Bursting pressure (mmHg) of fetal membranes in the study groups. Distributional plots express median and interquartile range, n = 8. Intact: intact membrane; Cyanoacrylate: positive control; S-DHA: silicone with dopaminated hyaluronic acid; SPU-DHA: silicone-polyurethane with dopaminated hyaluronic acid; S-HPMC: silicone with hydroxypropyl methylcellulose; SPU-HPMC: silicone-polyurethane with hydroxypropyl methylcellulose; ns: non-significant. Figure created using Stata 14.2.

Membranes sealed with either cyanoacrylate or integrated systems S-DHA, SPU-DHA, S-HPMC or SPU-HPMC showed median values of bursting pressures in the range of 16–48 mmHg, with no significant differences among groups (Fig. 2).

**Figure 3:**
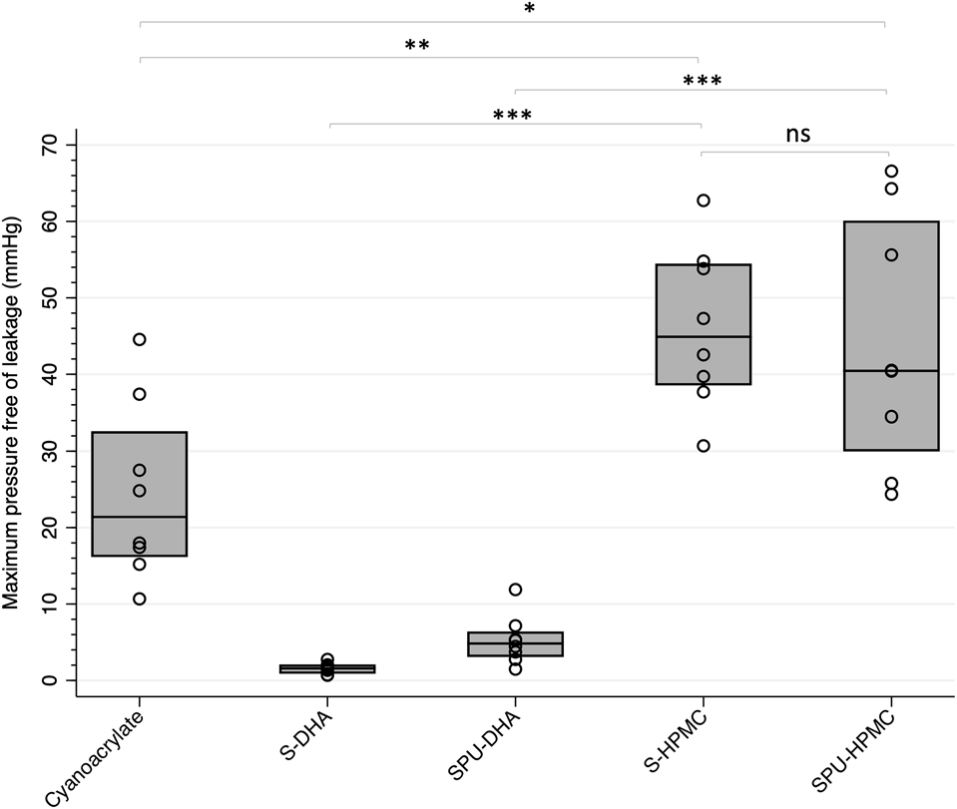
Maximum pressure free of leakage (mmHg) supported by fetal membranes according to treatment. Distributional plots express median and interquartile range, n = 8. *p < 0.05, **p < 0.01, ***p < 0.001. Intact: intact membrane; Cyanoacrylate: positive control; S-DHA: silicone with dopaminated hyaluronic acid; SPU-DHA: silicone-polyurethane with dopaminated hyaluronic acid; S-HPMC: silicone with hydroxypropyl methylcellulose; SPU-HPMC: silicone-polyurethane with hydroxypropyl methylcellulose; ns: non-significant. Figure created using Stata 14.2.

**Figure 4:**
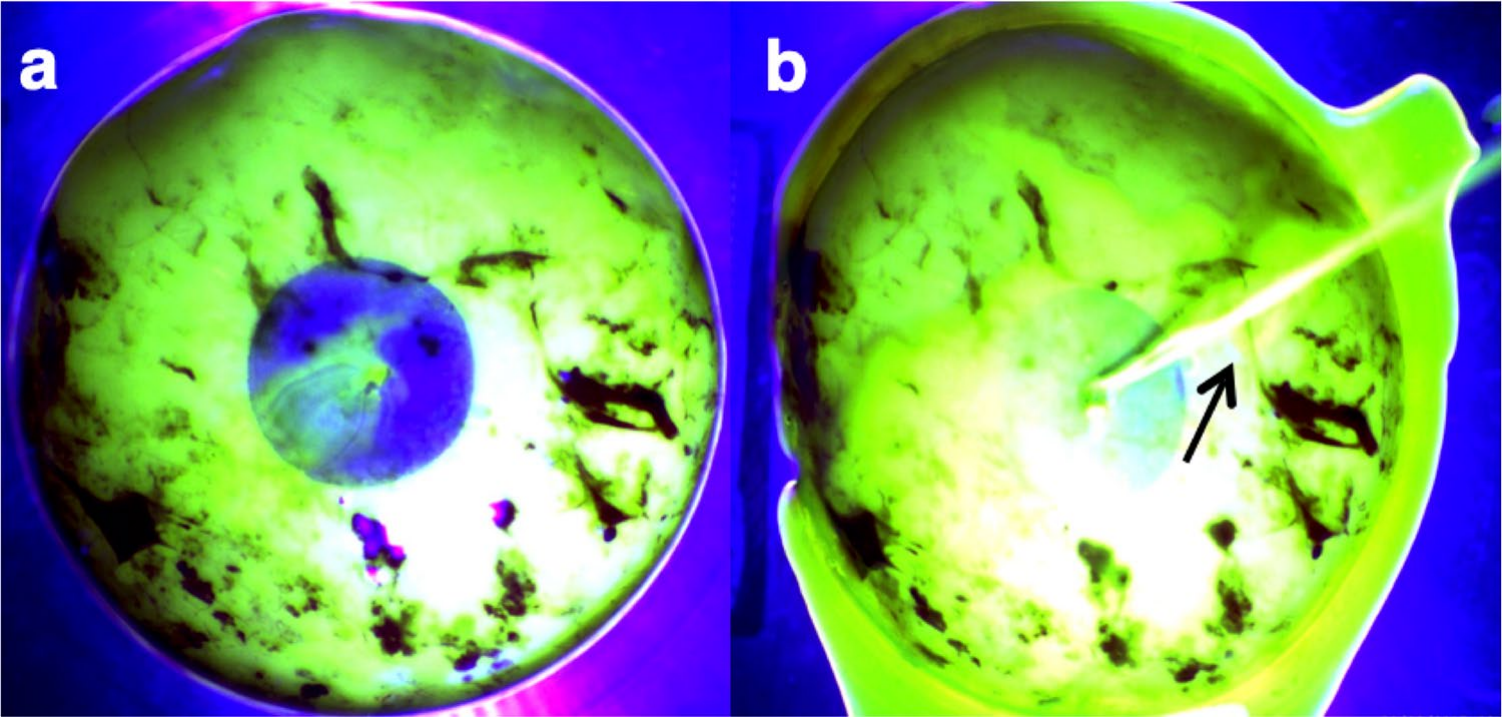
Representative images of leakage, rupture and completely sealed membrane. (**a**) Fluorescent light: leakage is observed as fluorescent flow through the lesion. (**b**) Fluorescent light: rupture of membrane characterized by a sudden decrease of internal pressure associated or not with an important fluorescein flow through the lesion (long arrow).

In terms of maximum pressure free of leakage (Figs. 3, 4), S- and SPU-DHA patches presented leakage at low pressures (medians ranging from 1.6 to 4.8 mmHg). On the contrary, S- and SPU-HPMC candidates showed leakage at higher pressures [S-HPMC 44.9 mmHg (IQR 38.7–54.3) and SPU-HPMC 40.5 mmHg (IQR 30.1–59.9)], which were significantly higher than those observed with cyanoacrylate [21.4 mmHg (IQR 16.3–32.4)], *p* = 0.005 and 0.02 respectively.

### Toxicity

**Figure 5:**
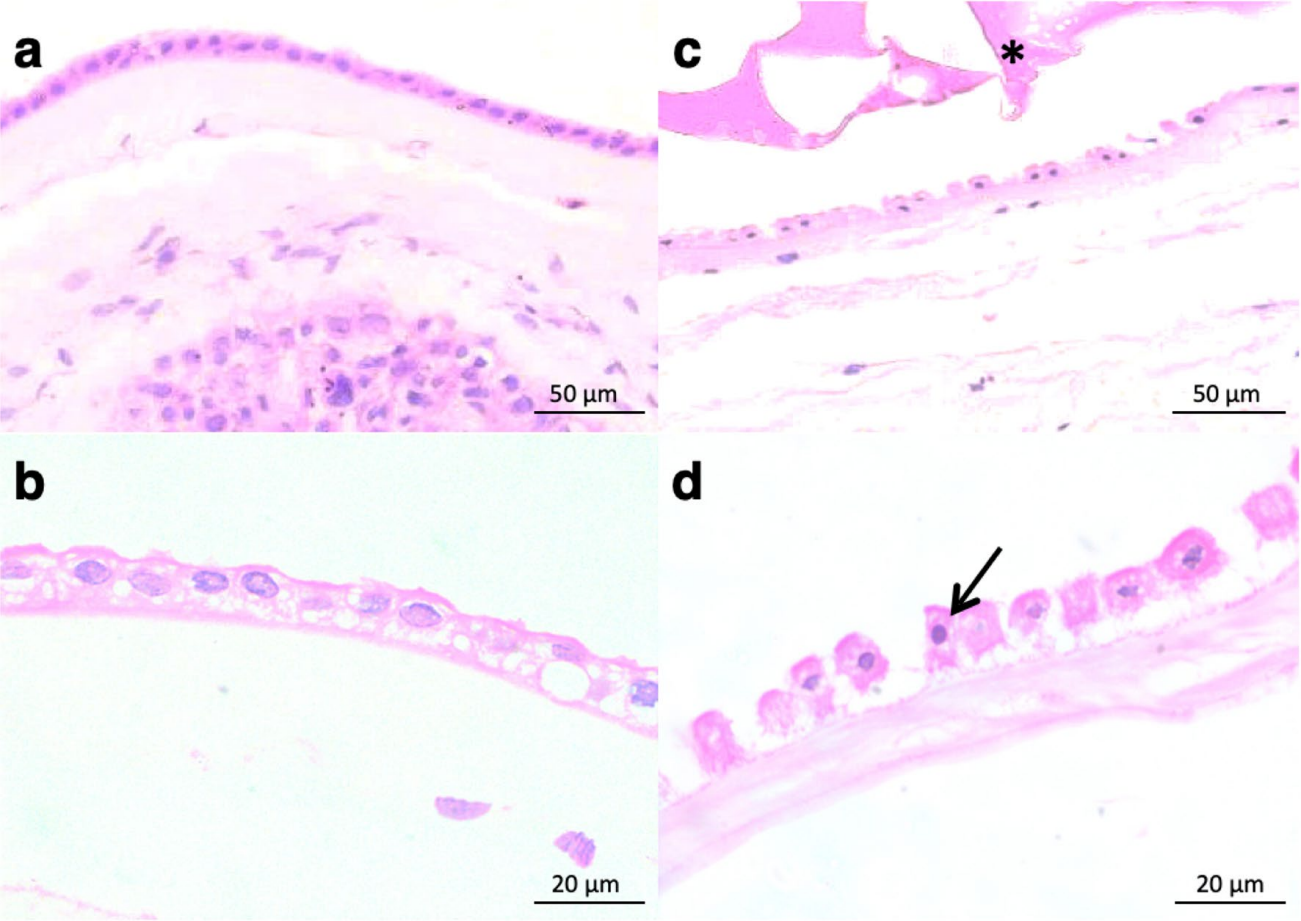
Histological assessment of toxic effects on fetal membrane amniotic layer (hematoxylin eosin) after 48 h of culture. (**a**,**b**) Non-exposed (NE) membrane at × 400 and × 1000 magnification, respectively. (**c**,**d**) Membrane exposed to positive control at × 400 and × 1000 magnification, respectively. In this group, pyknotic nucleus is identified (arrow). *Eosinophilic material corresponding to positive control.

**Figure 6:**
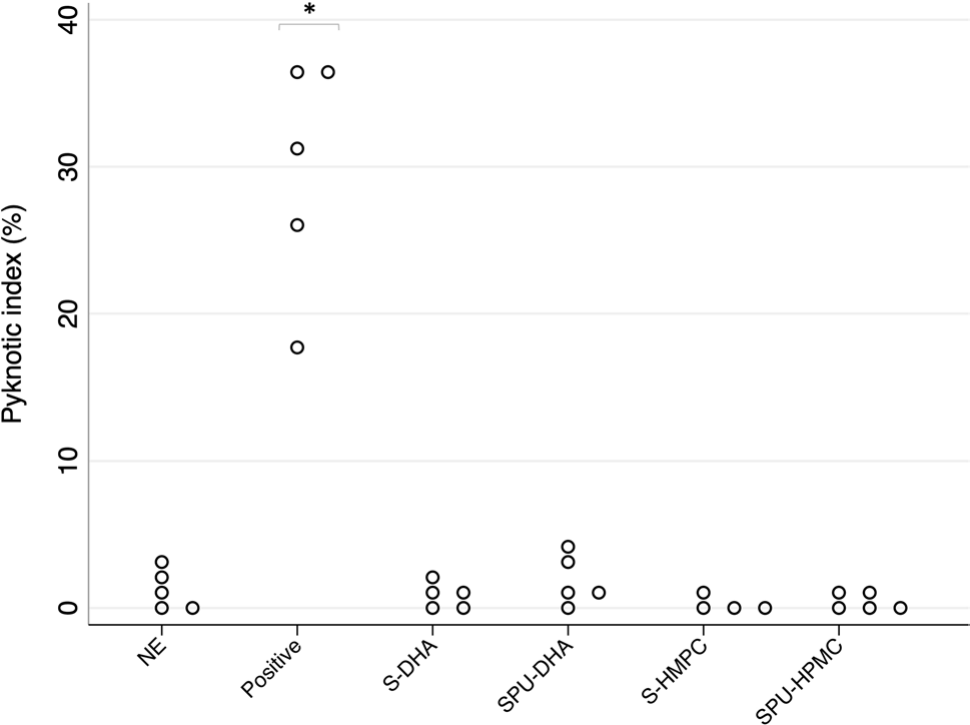
Pyknotic index of amniotic cells nuclei (in percentage) according to bioadhesive groups at 48 h of culture. Distributional dotplots, n = 5. *p < 0.05 (comparison between positive control and the other groups). NE: non-exposed cultured membrane. Positive: positive control (component B of DrSails glue); S-DHA: silicone with dopaminated hyaluronic acid; SPU-DHA: silicone-polyurethane with dopaminated hyaluronic acid; S-HPMC: silicone with hydroxypropyl methylcellulose; SPU-HPMC: silicone-polyurethane with hydroxypropyl methylcellulose. Figure created using Stata 14.2

Pyknotic index at 48 h of culture (Fig. 5) was on average below 2% in all bioadhesives tested and in non-exposed membranes (*p* = 0.483), while in positive controls with commercial adhesive it was 31.1% (IQR 26.0–36.5), *p* < 0.05 (Fig. 6).

**Figure 7:**
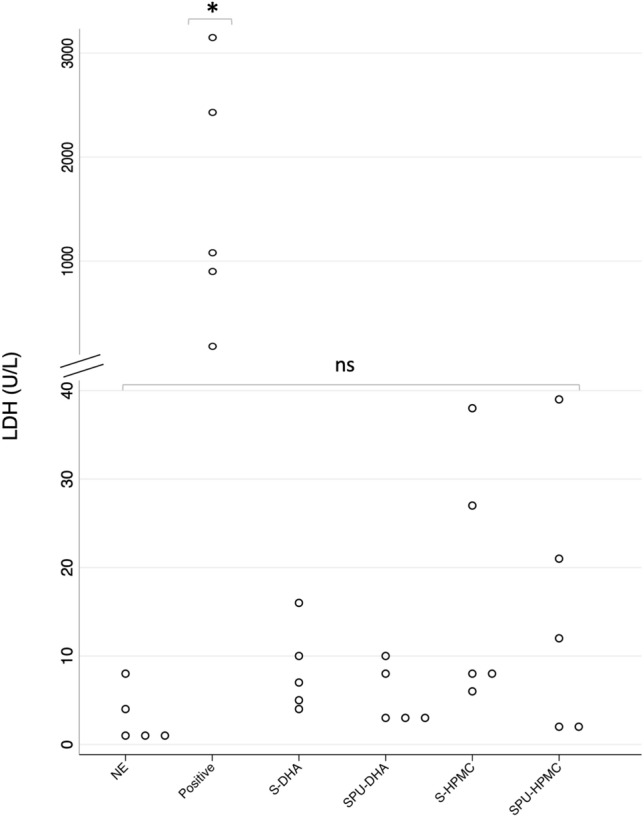
Concentration of lactate dehydrogenase (LDH) in U/L in supernatants according to bioadhesive group at 48 h of culture. Distributional dotplots, n = 5. *p < 0.01 (comparison between positive control and the other groups). NE: non-exposed cultured membrane. Positive: positive control (component B of DrSails glue); S-DHA: silicone with dopaminated hyaluronic acid; SPU-DHA: silicone-polyurethane with dopaminated hyaluronic acid; S-HPMC: silicone with hydroxypropyl methylcellulose; SPU-HPMC: silicone-polyurethane with hydroxypropyl methylcellulose; ns: non-significant. Figure created using Stata 14.2.

Likewise, median LDH concentration at 48 h of culture ranged from 3 to 8 U/L and was similar to non-exposed membranes (*p* = 0.154). The levels of LDH were significantly higher in the positive control 1754 U/L (IQR 998.5–2775.5), *p* < 0.01 (Fig. 7).

## Discussion

In this study we tested an innovative integration of a semi-rigid biocompatible patch coated with bioadhesive. The results demonstrated that such system can achieve effective sealing of a defect created in human membranes ex vivo, with similar adhesiveness to that achieved by cyanoacrylate, but without cell toxicity. These results open new possibilities for the development of membrane sealing systems aiming at reducing the prevalence of PPROM after fetoscopy.

Our sealing system is based on the concept of sealing fetal membranes from the amniotic side. This may offer advantages to previously tested solutions. Collagen-based plugs have shown negative results when used clinically^18,18^. Among other reasons, plugs can migrate, increase the size of the defect and they dissolve quickly in most instances^18,18^. In addition, a physical plug across membranes might interfere with the natural protection against PPROM provided by the sliding of the amniotic over the chorionic membrane layers^6^. Among fluid adhesives available for medical use, cyanoacrylate glues are extremely adhesive to tissues even in wet conditions. However, when applied as a glue, they show non-elastic adhesion with risk of damage due to traction^20^ and its application in intrauterine environment is complex due to its non-solid condition. Previous studies have investigated the use of mussel-like glues as an alternative to cyanoacrylate^15^, since they are based on a catechol side chain of 3,4-dihydroxyphenylalanine (DOPA) amino acid that theoretically secures strong adhesion to almost any surface even underwater^15,15^. Indeed, dopaminated-poly(ethylene glycol) (PEG), a mussel-like bioadhesive, was able to achieve leak-proof closure of 3.5-mm-trocar defects while stretched in vitro^15^ and has shown to withstand bursting pressures around 35–40 mmHg^13,13^. In general, the application of glues on amniotic side of fetal membranes in clinical conditions is challenging^13,14,22^. One study reported the development and ex vivo testing of an umbrella-like device supported by a purpose-designed mesh as a potential solution to apply a liquid mussel-glue to the amnion defect^17^.

In this study we tested different adhesive polymers. To achieve a system similar to the dopamination of mussel-glues, we produced DHA from the polysaccharide hyaluronic acid. DHA adhered initially to membranes in the multiaxial device and achieved bursting pressures of 16–48 mmHg, similar to those reported using other bioadhesives mainly based on mussel-glue-like systems^13,15,22^. However, DHA showed leakage from pressures as low as 1.6 mmHg. We decided to evaluate HPMC polysaccharide in the study due to its natural adhesive properties^23^. Contrary to DHA, HPMC supported high bursting pressures preventing leakage up to 40–45 mmHg. Regarding the composition of the semi-rigid patch, S and SPU are largely used in medical devices^24,24^. Since S is extremely hydrophobic and poorly adherent, polyurethane was added to tune the hydrophobicity. Moreover, such coating reduces the stiffness of the surface in contact with the tissue, making the materials mechanically friendly with the amniotic membrane. We demonstrated that both materials (S and SPU), coated with HPMC, had a good performance ex vivo. It is important to highlight that both S and SPU patches were produced with a micropatterning in order to increase the contact surface and to generate friction force. These features would contribute to improve attachment of the bioadhesive to a slippery surface such as the fetal membrane.

This study has clinical and research implications. Preventing iatrogenic PPROM is one of the major challenges in fetoscopic surgery. The system here presented has some potential advantages that would make it a suitable candidate for clinical testing. The use of a semi-rigid patch allows insertion through a fetoscopic trocar and eliminates the challenges of applying in an efficient and safe fashion a liquid glue on human membranes^17^. The biocompatible, non-absorbable nature of the materials used allows long-term use and prevents degradation in a biological environment, as observed with the use of other candidate systems for membrane sealing^15,15^. Research on the feasibility of application and efficacy of the bioadhesive patches in an experimental model of fetoscopic surgery is now under way.

Among the strengths of the study, we used an ex vivo model with real human membranes. The adaptation of a previously validated approach^13^^26,27^ with the addition of fluorescein, allowed detection of subtle leakage more objectively. Contrary to previous studies in which glues were tested over chorion or myometrium^13,13,13^, we applied the sealing system over the amnion, upside down and under a fluid environment, reproducing the conditions of a clinical application. Finally, we challenged substantially the system using pressures far above the range of 15–30 mmHg of human pregnancy^29,30^. Among the limitations, these ex vivo results require further validation in other systems evaluating long-term adhesion and feasibility in experiments simulating real clinical conditions. We used term membranes and although there are no remarkable histological differences with preterm membranes^31^, it could be argued that adhesiveness could be lower on the latter, especially due to differences in stiffness^32^.

In conclusion, an integrated semi-rigid patch using HPMC as bioadhesive achieved levels of adhesion similar to a well-known adhesive as cyanoacrylate glue, but with the advantage of composing a non-toxic and ready-to-use system. The results warrant further research to test long-term adhesiveness and feasibility as a sealing system for fetoscopic procedures.

## Methods

### Ethical aspects

All methods were carried out in accordance with relevant guidelines and regulations of the ethics committee. The experimental protocols were approved by the Ethical Committee of Hospital Clinic (HCB/2016/0236) and of Hospital Sant Joan de Déu (PIC-40-16). Informed consent was obtained from all patients, none of them under 18. Confidentiality and anonymity were ensured, and data was used only for scientific purposes.

### Fetal membrane collection

Fetal membranes were collected after written consent from elective cesarean sections of singletons at term (gestational age from 37 to 41 weeks). Exclusion criteria were: clinical chorioamnionitis, infections (HIV, hepatitis, syphilis), disturbances of amniotic fluid (oligo or polyhydramnios at the last scan), maternal conditions such as hypertension, diabetes, anemia, connective tissue disorders, under nutrition, use of tobacco, fetal growth anomalies, major fetal malformations or chromosomal abnormalities. Patients with history of contractions, preterm cervical dilatation or premature rupture of membranes were also excluded.

### Production of the patches and bioadhesive systems

The semi-rigid patches were produced using either medical silicone (S—NuSil, NuSil Technology LLC, United States) or medical silicone combined with electrospun polyurethane (SPU—NuSil and Bionate Thermoplastic Polycarbonate-Urethane, DSM Biomedical Headquarters, United States). Both patches were produced in a 17-mm diameter disc shape, from 450 to 480 µm-thick. The surface was prepared for receiving the bioadhesive component by producing a 150 µm deep micropatterning on it, to enhance contact surface and adhesion. All sealing systems were used up to 35 days after their production.

Both silicone (S) and silicone-polyurethane (SPU) disc patches were coated with bioadhesive components composed either by dopaminated hyaluronic acid (DHA) or hydroxypropyl methylcellulose (HPMC). DHA was used at 10 mg/mL and oxidized with ferric chloride, with 70–80% dopamination. HPMC was used at 10 mg/mL after plasma activation of S or SPU.

### Membrane processing

After collection, fetal membranes were separated from the placenta. Areas with visible clots or decidual contamination were removed. Specimens were transported to the laboratory for further handling in saline solution at room temperature within 30 min. Explants were cut at 2 cm from placental edge to avoid heterogeneity of the tissue^33^ and used within 6 h. In case the membranes were not used immediately, samples were kept at 4 °C until use.

A total of 27 fetal membranes were needed. For mechanical experiments, each sealing system and control was tested 8 times with membranes from different donors, to reduce the possible effects of membrane variability between donors. Each fetal membrane was used, in average, for 2–5 tests, depending on the conditions of the membrane. For toxicity, each experiment with sealing system and control was done 5 times with membranes from different donors, to reduce the possible effects of membrane variability between donors.

For mechanical properties, fetal membranes were prepared as follows. Explants were cut in 7 cm diameter circles with scalpel. Sand papers rings (waterproof paper, no 180, ADSEng, Germany) with 50 mm inner and 70 mm outer diameter were glued on both sides of the membrane with superglue (Super Glue-3 Original, Loctite, Barcelona, Spain). Samples were kept moist with phosphate buffered saline (PBS) during the whole process. Before mounting the fetal membranes over the inflation device, explants were perforated with a 10Fr-metalic punch (3.33 mm—Karl Storz SE and Co, Germany), from chorion to amnion (simulating clinical condition) in the center of the circumferential membranous explant. Perforated membranes were then sealed with the different sealing systems or a positive control (Cyanoacrylate). Sealing systems or positive controls were placed with tweezers over the amnion to cover the defect and adjusted with gentle pressure for 60 s. After 2.5 min, fetal membranes where then mounted over the cylinder with the amnion facing a fluorescein saline solution, as followed explained.

For toxicity experiments, samples were handled in sterile fashion and used immediately after collection. Membranes were cut in 6 fragments of 2 × 2 cm and applied in contact with the different experimental groups.

### Mechanical properties

A multiaxial inflation device was used to evaluate the mechanical properties of the sealed membranes. It consists of a custom-built device that was previously characterized by Haller et al.^13,13,13^ and provided to us by the Department of Mechanical Engineering of Swiss Federal Institute of Technology (Zurich, Switzerland). Briefly, the device consists of an aluminum cylinder with a 50 mm-inner diameter that was mounted by clamping membrane samples between the cylinder and a cover ring. The cylinder was connected to inlet and outlet tubes. A peristaltic pump (Reglo Digital ISM834C, four channels, max 35 ml/min per channel, Ismatec Laboratory Pumps, Wertheim, Germany) connected to the inlet tube was used to gradually fill the cylinder with fluorescein 0.1% prepared in PBS (Fluorescein oculos 10%, SERB Specialty Pharmaceuticals, Belgium). Fluorescein was used for detection of leakage out of the iatrogenic defect.

The cylinder was inflated by increasing the solution volume with a constant pumped rate flow of 2 ml/min. The difference of pressure generated was measured throughout the experiment with a hydrostatic pressure sensor (digital manometer, LEX 1, − 1 to 2 bar, accuracy within 0.05%, Keller, Switzerland) positioned at the outlet tube and connected to a computer with a converter (K-114 A USB to RS485 Converter, Keller, Switzerland). An UV flashlight (UV light 400 nm 51-LED flashlight) was used to improve detection of fluorescein leakage. The deformation of the membrane was monitored with a video camera (CMOS Camera 1280 × 1024, Color, USB2.0, 18-108 mm EFL Zoom Lens w/Focus Control 2/3″ format, Thorlabs, Munich, Germany) mounted on the top of the cylinder.

Intact membranes (without perforation) and membranes sealed with an industrial cyanoacrylate glue (2-ethyl cyanoacrylate 75%, 1,4-dihydroxybenzene, hydroquinone, quinol—Superceys Unick, Ceys S.A., Barcelona, Spain) were used as controls. Cyanoacrylate glue was added manually to 17-mm diameter disc-shaped silicone patches a few moments before the test and therefore did not compose an integrated system.

Mounted membranes were inflated by continuously increasing the pressure in the cylinder until rupture of the membrane. Rupture was characterized by a sudden decrease of internal pressure being associated or not with an important fluorescein flow through the lesion. Bursting pressure was registered. The occurrence of leakage before bursting pressure was defined as detection of fluorescein flow through lesion while the pressure was still increasing and the membrane still inflating. Maximum pressure free of leakage was also registered.

### Toxicity

The toxicity test was performed as previously reported^8^. Under a laminar airflow hood, the membrane explants were washed in sterile PBS (PBS pH7.4-1x—Gibco by Life Technologies, USA) at 37 °C. Areas with visible blood clots or decidual contaminations were removed. The explants were mounted over an acellular collagen support (Lyostyp—B. Braun, Germany) in 12-well plates, with the chorion facing the collagen support.

Different sealing systems and controls were placed on the amnion surface of fetal membrane fragments and incubated with a complete culture medium for amniocytes (Amniomax—Complete Medium—Gibco by Life Technologies, USA) at 37 °C and 5% CO_2_ air up to 48 h. Initial experiments with cyanoacrylate showed remarkable tissue destruction. To achieve tissue integrity allowing histological evaluation it was decided to replace cyanoacrylate by another commercial adhesive that also works in underwater conditions (DrSails—Sailing Technologies, S.L., Spain—component B, composed of modified polyamine). Tissue and supernatants of each group were harvested at 48 h. The groups studied for toxicity were: non-exposed membranes (cultured membranes without treatment), positive control (cultured membranes with 1 ml of component B of DrSails glue—Sailing Technologies, S.L., Spain) and sealing systems S-DHA, SPU-DHA, S-HPMC and SPU-HPMC (cultured membranes with sealing systems).

Tissue samples were fixed in 4% formalin for 24 h and embedded in paraffin. Transversal sections were obtained (4 µm) and stained with hematoxylin–eosin (HE). Overall morphological condition of the membranes was examined and pyknotic index, which is a characteristic feature of apoptosis^35,35^, was determined in the amnion layer to evaluate toxicity. Pyknotic and non-pyknotic nuclei were counted under light microscopy at 40-power magnification level (total magnification × 400) in 5 non-overlapping fields. Pyknotic nuclei were identified as small-sized nuclei and with highly condensed chromatin^37^. The pyknotic index was calculated by determining the percentage of pyknotic nuclei over the total number of amniotic nuclei.

Supernatants were stored at − 20° and used for quantitative determination of cytotoxicity based on quantification of lactate dehydrogenase (LDH) enzyme released into culture medium (ADVIA Chemistry Systems Lactate Dehydrogenase L-P (LDLP) assay, Siemens Healthcare S.L.U, Spain). Absorbance was read at 340/410 nm.

### Statistical analysis

Categorical variables were expressed as number of cases out of total and proportion (%). Normal distribution was verified using standardized normal probability plots, box plot graphs and Shapiro–Wilk normality test. Parametric data was expressed as mean ± standard deviation and non-parametric data was expressed as median (interquartile range). Homogeneity of variances was verified with Levene’s test. Inferential analysis for numeric non-parametric variables was performed using Kruskal–Wallis test with Bonferroni error correction or Wilcoxon rank test (Mann–Whitney). Statistical significance was defined as *p* value < 0.05 for all analysis. Data was processed using Stata 14.2 (StataCorp. 2015. Stata Statistical Software: Release 14. College Station, TX: StataCorp LP).

## Data Availability

The datasets generated during and/or analyzed during the current study are available from the corresponding author on reasonable request.
